# Emotional Testimonies: An Ethnographic Study of Emotional Suffering Related to Migration from Mexico to Arizona

**DOI:** 10.3389/fpubh.2015.00177

**Published:** 2015-07-13

**Authors:** Rebecca Crocker

**Affiliations:** ^1^School of Anthropology, University of Arizona, Tucson, AZ, USA

**Keywords:** mental health, emotion, depression and anxiety disorders, stress, Mexican immigrants, structural vulnerabilities, embodiment theory

## Abstract

It is increasingly argued that social and economic inequities poorly affect overall health. One of the means through which these inequities are translated to the body is via negative emotions, which carry known psychological and physiological responses. This paper examines migration-related psychosocial stressors impacting first-generation Mexican immigrants in southern Arizona, and reports on the primary emotional experiences immigrants associate with these stressors. Data were drawn from a qualitative, ethnographic study conducted over the course of 14 months during 2013–2014 with first-generation Mexican immigrants (*N* = 40) residing in Tucson Arizona and service providers working directly in the immigrant community (*N* = 32). Results indicate that the primary structural vulnerabilities that cause emotional hardship among immigrants are pre-migration stressors and adversity, dangerous border crossings, detention and deportation, undocumented citizenship status, family separation, and extreme poverty. Many of these factors have intensified over the past decade due to increased border security and state level anti-immigrant legislation in Arizona. Immigrants connected these hardships to the emotions of trauma (50%), fear (65%), depression (75%), loneliness (75%), sadness (80%), and stress (85%), and most respondents reported suffering from three or more of these emotions. Given the heavy emotional toll of migration and the direct impact that regional legislation and border security had on well-being, this paper argues that emotion be considered an important mechanism for health declines in the immigrant community. In order to stem the frequency and intensity of emotional stress in the Mexican immigrant community in Tucson, it is imperative to support organizations and policies that promote community building and support networks and also expand access to and availability of mental health services for immigrants regardless of documentation status.

## Introduction

This article aims to establish emotion as a critical means by which the context of immigrant life gets transferred to the bodies of individual Mexican immigrants. As the means by which individuals sense the structure of their larger environs, emotion can be understood to link our bodies to the outside world (1). Based on a qualitative, ethnographic study among first-generation Mexican immigrants in southern Arizona, this article highlights the contextual factors that Mexican immigrants commonly connect to emotional suffering, namely pre-migration stressors, dangerous border crossings, undocumented citizenship status, detention and deportation, family separation, and extreme poverty. I then use the words of the immigrants themselves to reveal the nuanced marks of stress, loneliness, fear, sadness, and trauma related to these factors. Emotional suffering often remains masked by the individual and disassociated from both the structural causes that contribute to it and from its broad and debilitating impacts on the body (2). By drawing intimate emotional experiences out of the realm of the secret body and into the light of critical academic analysis, this article seeks to expand the discussion on structural causes of health disparities and identify policy recommendations specifically geared toward reducing emotional stress.

This article employs the theoretical framework of structural vulnerability to examine how the daily “lived lives” (3) of Mexican immigrants in southern Arizona may generate emotional suffering. Quesada et al. argue that Mexican immigrants have come to “occupy a disjunctive liminal quasi-caste status” in which they are marginalized via processes of economic subordination, cultural depreciation, and legal persecution, all of which has left them structurally vulnerable to poor health [(4), p. 346]. While the most commonly identified structural barrier to immigrant health is limited access to medical care (5), a smaller body of literature has documented the occurrence and potential health consequences of other powerful psychosocial stressors present during immigration. These include discrimination (6); targeted enforcement by police and border authorities (7, 8); social isolation (9); mistrust of police authorities and fear of deportation and detention (10, 11); and family separation (12).

The presence of social suffering and stress related to contextual circumstances is increasingly being documented in the literature on marginalized groups. Yet, we know far less about the nuanced, day-to-day, and highly individualized emotional impacts of these experiences [see Ref. (2, 3, 12–14)]. Krieger (15) states that disentangling the causal mechanisms by which our bodies express and mirror our social environments is the key to future health studies (16–18). The phenomenological theory of embodiment holds that the body is in constant dialog with its surroundings and relationships (15, 19–21), and it follows that immigrants carry the intimate imprints of migration-related stressors in their physical bodies. In fact, periods of physical displacement, such as migration, likely produce intensified embodied consequences, because “geographic orientation is embedded in the whole body” [(22), p. 1518]. As such, migration may be experienced by Mexican migrants as a fundamental ungluing, a disembedding, and reembedding of the body into unfamiliar and often hostile spatial and social worlds.

The potential contribution of this research on migration-related emotional suffering lies in the wide-ranging health impacts of emotion. While the concept of emotion remains a contested concept in social theory (1), emotional suffering and stress carry known psychological and physiological responses with important consequences for overall health (23–25). It is increasingly argued that inequitable structural factors in society produce health disparities (26, 27). Using the theories of structural vulnerability and embodiment, this study highlights emotional experience as a key mechanism by which the marginalization of immigrant life gets translated to the bodies of individual Mexican immigrants. Given that the well-documented health declines that occur in the Mexican immigrant community post-migration remain poorly understood (28, 29), emotion offers an important means by which to explore how race and citizenship status “get under the skin” to impact the mental and physical health of Mexican immigrants (30).

## Materials and Methods

### Research setting

Lying just 70 miles north of the current US–Mexico border, Tucson, AZ, USA is a rich setting for exploring the emotional experiences of migration. The city and surrounding areas house a large and diverse population of Mexican immigrants ranging from recent arrivals to established Mexican American families pre-dating the transfer of the region to American jurisdiction in 1854. According to the 2010 U.S. Census Bureau, 41.6% of Tucson’s population was of Latin American origin and over a quarter of that population was foreign born. Statewide in Arizona, over 10% of public school students currently have at least one undocumented parent (31), further demonstrating the high percentage of Arizonans living in immigrant and mixed status families. Tucson’s historical origins as a Mexican town and its enduring status as an immigrant receiving community are reflected in the city’s wealth of Mexican storefront specialty shops and markets, cultural celebrations, historical landmarks, and bilingual community services. Yet, a longstanding and prejudicial “racial subtext” (32) underpinning Arizona state laws can be traced back to the state’s founding and has climaxed over the past decade as Arizona has witnessed a dramatic spike in immigration from Mexico during a period of state recession (33). This surge in-state level exclusionary legislation has had very real and deleterious effects on immigrants’ ability to access recreational, religious, educational, and health services (34).

### Study sample and procedure

This qualitative ethnographic research project was conducted over the course of 14 months in 2013–2014. Project approval from the University of Arizona Institutional Review Board (IRB) was obtained in November 2013 and extended for a second year in September 2014. The exploratory phase of research consisted of semi-structured background interviews (*N* = 32) with local activists and service providers selected on the basis of snowball sampling techniques in addition to extensive research into immigrant-serving organizations. The interview guide was designed to draw out information on conditions of daily life for Mexican immigrants, impact of state laws on the provision of medical services, structural vulnerabilities in immigrant life, and group-level health risks and outcomes. This exploratory research helped to establish the demographic parameters of the sample population, inform the immigrant interview guide, and identify appropriate venues to reach this vulnerable community.

I then conducted participant observation and in-depth semi-structured interviews (*N* = 40) with first-generation adult immigrants from Mexico. Because randomized techniques, such as phone sampling or recruitment of interviewees in public spaces, were not feasible or advisable given immigrants’ high rate of mobility and their concerns about avoiding detection by police authorities, I employed the method of “venue-based application of time-space sampling,” a convenience sample strategy using known venues where members of hidden populations gather safely (35). The sites identified for this study include a day laborers’ center, a women’s empowerment group, and two free medical clinics that serve the undocumented and uninsured communities. Due to the extreme vulnerability of undocumented individuals, study participants were consented verbally in lieu of signing a written statement. I recorded the interviews using a digital audio recording unless background noise was too distracting, and I also took copious notes regarding non-verbal cues to emotion. These precautions helped establish a safe environment for potential participants and only one person I approached for an interview declined to participate.

This study employed a life-history approach to immigrant emotional health, addressing factors intersecting with the emotional health of individual migrants before, during, and after the process of migration (36). Questions addressed early life in Mexico, and pre-migration stressors including childhood poverty, nutrition, emotional trauma, illness, and access to health care in order to establish a binational approach to migrant health capable of highlighting intra-group variations (37). Large-scale studies linking childhood exposure to poor health outcomes in adults make clear the necessity to examine health risks faced by migrants before arriving to the U.S. Though such studies lack inclusion of migration-related risk factors (8), a small body of qualitative research has demonstrated that variations in health exposures in Mexico have important ramifications for health behaviors including diet and pursuit of health care once in the U.S. (38–41). The life-course approach also highlights the increasingly lengthy and dangerous border crossing, which should be considered a health determinant in and of itself (42). Since the topic of illegal border crossing can be sensitive, I approached the issue by asking: “What mode of transportation did you use to reach the United States?” which enabled participants to address the experience of the crossing and related dangers and stressors.

I employed a fixed interview guide, which included a brief survey section tabulating basic demographic information on family background and physical and mental health problems. The majority of the interview addressed the structural challenges faced by immigrants once in Tucson, including those mentioned in the introduction as well as experiences of discrimination, barriers to healthy diet and exercise, and lack of access to medical care. I then presented participants with a list of both positive and negative emotions and asked that they identify those they related most strongly to their experience of immigration. I followed up on these response with tailored questions such as “how did you cope with being in jail during those weeks?” or “When the bills come in, where do you feel that stress in your body?” or “When your son was deported, how did that change your feelings about living here?” The interview guide also covered definitions of health, disease etiology, and healing traditions and practices pre and post-migration.

I conducted this research in Spanish, relying on my own professional training as a Spanish language interpreter and translator, as well as the assistance of native speakers and dictionary sources when necessary. I conducted the complete interview guide with all 40 participants, and interviews ranged in length from 1 to 3 hours. In addition, I conducted participant observation in courtrooms, political protests, churches, and participant’s homes in order to gain intimate familiarity with the structural and social factors shaping immigrants’ daily lives. My regular weekly appointments as volunteer English teacher and medical interpreter also enabled more extended and informal interactions with approximately one-quarter of sample participants who regularly attended either the health clinic or day laborers center where I worked. For the protection and privacy of all who participated in this study, I use pseudonyms throughout this article.

### Analysis

Data analysis was an iterative process that spiraled back and forth between data gathering and examination during the course of the project. Using MAXQDA qualitative data analysis software, I first coded the service provider interviews using “focused coding” methods designed to identify salient themes in immigrant life. I then simultaneously transcribed and translated the immigrant interviews, and later coded them according to the principles of “selective coding” (43), highlighting the links between categories of codes addressing the most common migration-related hardships and their associated emotional responses, in order to define the relationship between migration-related stressors and declines in health and well-being in individuals.

## Results

The Section “Results” of this article is organized according to the six primary emotional stressors identified by study participants: pre-migration stressors, dangerous border crossings, undocumented citizenship status, detention and deportation, family separation, and extreme poverty.

### Demographic portrait of study population

The study sample was evenly divided between men and women, with a median age of 42 (Table 1). This study sample included mostly long-term stable residents of Tucson, with the median number of years spent in residence being 15. Participants were evenly divided between having been raised in rural versus urban environments in Mexico. Just over one-half of this sample population was from Arizona’s bordering state of Sonora. Other highly represented states were Sonora’s southern neighbor Sinaloa and two southern states, Oaxaca and Chiapas, while remaining states of origin included Veracruz, Distrito Federal, Michoacán, Jalisco, Chihuahua, Guanajuato, Zacatecas, and Puebla. This sample reflects the recent diversification of the historically Sonoran-origin Mexican population in Tucson in response to shifting patterns of emigration from Mexico as well as the purposeful funneling of migrants through the deserts of Arizona (33).

**Table 1:** **Study sample demographics**.

Factor	Men (*n* **=** 20)	Women (*n* **=** 20)
Median age	38	43
Median residency in US (years)	14.5	15
Of Sonoran origin (%)	60	55
Monthly income ($US)	1500	1100
Average number of household members in US	3.9	3.6

### Childhood adversity

The majority of participants came from large, working class families with the average number of children in the family being just over 6. Most cited economic scarcity in their childhoods, and many noted common experiences of lacking money for basic clothing and enduring periods of nutritional deprivation. Less than one-quarter of the sample completed high school, with most having left school in order to augment family resources through informal employment. Many participants cited beginning to work at a very young age, sometimes selling gum or cleaning cars out on the streets. While the majority of participants cited being healthy as children and having little need for medical intervention, less than half of participants had health insurance during their childhoods in Mexico, and most reported relying primarily on home remedies and traditional modalities for their non-emergency medical care. Eighteen percent of participants came from families in which one or more siblings died during infancy.

Despite these common economic challenges, participants largely described their childhoods in a positive light, recalling the warmth and safety of their extended family networks. When extreme deprivation, emotional trauma, or abuse was present, however, this delicate balance was upset, and several participants demonstrated lasting damage from childhood suffering in their adult life in the US. Approximately, one-fifth of participants reported this degree of childhood suffering, with the most common experiences cited being parental abandonment, domestic violence either in childhood homes or adult romantic relationships, severe material deprivation, and sexual abuse. Those participants who reported childhood trauma also reported on-going suffering from emotional imbalance and depression at the time of the interview.

Enrique is a 54-year-old man from the state of Zacatecas who grew up in dire poverty in a family of 15 children, only 6 of whom survived their first year of life. Although he is now a citizen and once had stable employment, Enrique’s health problems have steadily worsened, and he is now physically disabled and severely depressed. He believes his current state of ill health results from having started to work at age 11, often under very harsh and exploitative conditions in both Mexico and US. He said:

All of this comes from my childhood. I never knew what it was to go from childhood to adolescence. I went from childhood straight to adulthood, to the responsibility. And when you see other young getting to enjoy life, you say ‘why didn’t I ever get to do that?’ But we came from a very poor family. If we ate breakfast, we didn’t know whether there would be any more food that day, because my father didn’t have that awareness that he had to work in order to feed his children… So I had to leave school and play the role of father.

These pre-migration emotional and physical risk factors underscore the importance of employing a binational and life-course approach to immigrant health studies.

### Border crossing

Slightly over half of the participants crossed the border without legal permission, many of them crossing two or more times (Table 2). More men in the sample crossed without legal permission than did women (80 and 25%, respectively). Likewise, participants from Sonora had greater access to temporary tourist visas and thus were more likely to have crossed legally. Most study participants who immigrated without legal permission crossed on foot through the Arizona desert, reflecting the impacts of the federal “Border Strategic Plan” in the 1990s that shut down traditional points of entry in Nogales, San Diego, and El Paso (44). The majority of study participants who crossed the desert spent between $1,000–3,000 for a “*coyote*” to guide them and walked for several days. A nurse who regularly treats distressed migrants concludes that the stressors of crossing the desert on foot are so intense, that “if migrants have crossed the desert, their emotional health is very affected by that experience” (Roberts, personal communication).

**Table 2:** **Migration-related conditions and experiences (%)**.

	Men (*n* **=** 20)	Women (*n* **=** 20)
Undocumented residency status	60	75
Detained or deported	65	45
Separated from immediate family	45	35
Extreme financial hardship	80	75
Crossed through desert	80	25

Most desert crossers in this sample referenced the intense physical exertion, extreme temperatures, and bodily injury endured during the crossing period, which often resulted in dehydration and hunger. Aid workers in the desert reported that common injuries include blisters, sprained and broken lower extremities, cuts and scratches, snake bites, exposure to the elements, and emotional trauma due to assault and near-death experiences (Price and Wallin, personal communications). Serious bodily injury, such as acute kidney failure and broken bones, are likewise not uncommon.

Fear and trauma were the most commonly cited emotional experiences related to the crossing, which immigrants identified as a period of extremely heightened anxiety. Participants reported fear of criminal extortion and kidnapping, and several cited attacks and robbery by “*bajadores*” – Mexican criminals who assault migrants just before they cross the border. Once in US territory, participants feared detection by Border Patrol agents and Mexican drug runners, as well as non-human surveillance and threats to safety, such as animals, aerial drones, and ground sensors. Recovery from the emotional and physical stress of the border crossing was often slow. Lalo, a 37-year-old native of Chiapas, has crossed the border and been deported multiple times. He described the bodily sensation of crossing in this way:

When you are crossing, you have one 100% adrenaline; you are alert the entire time. If you get a spine in your body or are bitten by a spider, you don’t feel anything, your blood is hot, your mentality is to be totally alert. So at the time you don’t feel anything, but the problem starts after you arrive and you relax. I remember that I was taking spines out of my body for many days, like I didn’t feel all the scrapes and cuts for a long-time afterward.

Between 1990 and 2012, approximately 2,238 migrants are believed to have died in the Arizona deserts (45). Crossers often lack the control to improve their own conditions or help others in their group, and traumatic experiences therefore cause feelings of helplessness, anxiety, guilt, and deep sadness. Jacobo Tellez of the Mexican Consulate in Tucson recalls the day a boy faced 15-year-old desert crosser from Oaxaca was placed into the custody of the consulate after his father fell dead in the desert. After offering the youth whatever comfort they could, he said he was ready to tell his mother the news. “I connected the phone line and went to sit back down,” Tellez recalls. “But as soon as the boy heard his mother’s voice on the phone, he started wailing and screaming: ‘Mamá! Mamá! He died on me, I couldn’t do anything! I couldn’t do anything!’ and the pain in his voice ran chills through my body, because it was his sheer impotence, his inability to do anything for his father.”

Moreover, the perils of the desert crossing reverberate far beyond the individual crossers and into the immigrant community more broadly. Many participants described periods of high anxiety and worry as loved ones were attempting the desert crossing, with some having been extorted by criminals in exchange for the release of abducted crossers. A small minority of the sample had experienced the death of friends or family during the crossing. Elena Burgos, who offers “healing touch” massage therapy in Tucson, reports that many families come to her because of trauma related to the desert crossing. Burgos can relate to others’ experience because her own brother died crossing the desert.

Someone called my mom and said the animals had eaten [my brother’s] body and that they just found the bones, and that is when she had a heart attack. And we felt like we were losing our minds, because of course we never thought this would happen – we kept thinking that he was coming, that he would arrive. He had five kids and was 32 years old. So you feel like your heart is just breaking to pieces.

### Legal status

The majority (67.5%) of the sample was undocumented, 22.5% had work permits or were legal residents, and 10% were citizens (Table 2). There was not always a direct correlation between the illegal border crossing and current status an undocumented immigrant. Several participants who initially had to trek across the desert had since been able to achieve legal status. Contrastingly, almost half of the Sonorans in the study sample had initially crossed the border legally with a tourist visa, but had since out-stayed the visa and were undocumented. The majority of service providers and immigrant participants independently noted the damaging impacts of Arizona state legislation, specifically State Bill 1070, in increasing the dangers, frustrations, and stressors of undocumented life in Arizona. These compounding hardships in the lives of undocumented immigrants cause such emotional upheaval that Cavazos-Rehg et al. call undocumented status itself “a persistent and insidious psycho-environmental stressor” (2007: 1126). Participants with temporary work visas and permanent residency likewise expressed feeling vulnerable both to the whim of local and federal politics as well as that of individual law enforcement agents.

When I asked Abelardo, a 54-year-old day laborer from Chihuahua, how it felt to be undocumented in Arizona, he took off his worn sports cap, put his arm down on the table to rest his head and began to quietly sob. “It feels so hard,” he managed softly. “It just feels so bad.” Undocumented participants reported that their lack of legal status obstructed their ability to live “*a gusto*” or at ease, and caused emotional expressions including fear, sadness, trauma, frustration, helplessness, and loneliness. Due to the fact that Tucson houses the overlapping jurisdictions of local police forces, country sheriffs, Border Patrol, and Immigration and Customs Enforcement agents (ICE), fear of arrest and deportation was a very common experience for participants (65%). Many participants lamented the feeling of having to always “*andar muy recto*” (not make any mistakes) and to leave home only for work and other mandatory activities, often causing them to bypass necessary medical attention. Diana, who has lived in Tucson for 15 years without legal status, suffers from an autoimmune disease called pemphigus, the onset of which has been linked to emotional stress (46). She states:

For me, fear has been the most direct impact on my condition, what I suffer from. Fear of leaving the house or not returning, that at any moment I could be captured and identified as what we are, as immigrants. Even though I have an application in through my mom, it is still not a legal condition, so I am still dealing with the fact that I am here in limbo, without being anybody, without being a true entity as we say, a somebody.

Many participants described how their fear of police detection, particularly following the passage of SB 1070 in 2009, prevented them from participating in community activities and from fulfilling civic duties, such as reporting crimes in their neighborhoods. Moreover, their lack of legal residency and the dangers of the crossing made returning to Mexico virtually impossible (see Family Separation). This isolation in turn generated feelings of loneliness, which was a salient theme in the lives of 72.5% of study participants. According to Leticia, a 34-year-old married woman from Guanajuato:

I don’t have a lot of community – I think that’s because of the laws they’ve passed here. I feel like at any moment they can decide that we all need to leave. So my life, I feel like today it is good, but tomorrow I don’t know how it will be. That is why I started to feel depressed, because I feel like in reality I don’t have a stable life here. I feel part of Tucson but sometimes, like when I have to present an ID, my reality hits me and makes me feel like, no, I am really not a part of it.

Undocumented status likewise impeded immigrants’ ability to progress toward their goals, leading to feelings of hopelessness and frustration in regards to their educational and vocational opportunities. Many participants used words such as *estancado* (stuck) and *impotente* (helpless or hopeless) to describe their day-to-day feelings about being undocumented. Adrián, a 24-year-old who was brought from Sonora by his family as a small child recalled that: “In school growing up I always wanted to continue my education. But when I understood more of what it meant to be undocumented, it brought me down and I felt hopeless. I thought “why the heck should I go to high school if I can’t go to college?” I felt depressed and in my senior year I lost all motivation and almost dropped out.”

### Detention and deportation

Forty-five percent of women and 65% of men had faced experiences of detention, deportation, or both in their immediate families (Table 2). Undocumented immigrants were most commonly detained during the initial crossing, on routine traffic stops, and workplace raids. One participant was arrested in the process of reporting a crime to police. Deportation and detention carried acute emotional responses including trauma, loneliness, fear, and sadness, both for the person directly involved as well as for family members who faced concomitant emotional and financial challenges. Service providers also noted the impact on the wider community, as “the community is always fluctuating and is really unstable with detentions, deportations, and loss of jobs. This is really challenging for people without papers – there is an emotional tax in seeing people here one day and simply gone the next” (Sharer, personal communication).

Many participants reported that their fear and anxiety levels increased following their own deportation or detention or that of a loved one. Yesenia, a 42-year-old undocumented immigrant mother from Sonora, said that her brother’s deportation changed her daily experience in Tucson:

After my brother got deported six years ago, my fear got worse and I got sick from all the anxiety. It was like psychological terror. I can’t sleep until everyone in my family is altogether – I worry that one of them won’t come home one day. The last time the police pulled us over, I shook the policeman’s hand and said ‘*felicidades*. If what your government wants is to make us sick and keep us scared, then congratulations, you’re doing your job.’

Other participants described the sadness and loneliness that resulted from losing family members who had been deported and were unable to return. Teresa’s husband of 28 years was deported one day without notice, leaving her struggling to raise their teenaged son alone. Since his departure, Teresa has been evicted from their apartment and has fallen into a deep depression.

When he first left there were many days that I hardly ate, could barely sleep. I am embarrassed to say this but there are still days when I don’t even want to bathe myself or get dressed. It seems like no matter what I am doing, if I am on the bus, or walking somewhere, or going to the market, that I start to cry. And it seems like wherever I am that everything is ugly now. Even my plants, which used to be so beautiful to me, seem ugly now.

Detention also led to extended periods of separation, which were exacerbated because undocumented family members were unable to visit the jail due to their own lack of legal status. Eva, a 42-year-old who has lived in the US for over 15 years recalls the isolation of her 60-day separation from her then 4- and 7-year-old sons while in detention following a traffic violation. “It is so hard, imagine how lonely and sad you feel, without anyone there to accompany you. And on the weekend everyone else had visitors, but my sons and my husband couldn’t be there.” Study participants who had been detained also cited common experiences of discrimination and mistreatment, including overcrowded cells, freezing temperatures, lack of proper nutrition, intentional family separation, and denigrating treatment by guards, all of which has been extensively documented (7, 47).

According to a medical doctor who works in a federal prison in Arizona, the combination of these factors leads to toxic levels of stress and anxiety while incarcerated, which carry potential physical consequences including high blood pressure and blood sugar levels (Olsen, personal communication). Lalo recalled the desperation that set in soon after his arrival to a federal detention center in Eloy following a routine traffic stop in Tucson:

The doctor asked me if I suffered from schizophrenia, if I heard voices, or if I had tried to kill myself, and it seemed weird to me, because I have never wanted to do any of those things. But then I understood that the problem was when they locked us up, when they closed the door. And the guard yelled “don’t move, don’t make noise, go to sleep now!” And I started to feel desperate, my heart beat like crazy, and I felt claustrophobic and I started to sweat, and I wanted to throw open the doors and just fly out of there or disappear. I even wanted to hurt myself so they would take me out of there.

### Family separation

While participants mourned many aspects of what they missed about Mexico, including traditional foods, their personal freedoms, and the more relaxed pace of life, the vast majority cited family separation as the most emotionally challenging part of leaving Mexico. The primary emotions immigrants associated with these separations were loneliness, frustration, and sadness, which sometimes led to longer-term depression. Forty percent of participants were separated from a child or spouse for at least 1 year during their immigration experience, and the vast majority faced on-going separation from siblings and parents (Table 2). This high occurrence of family separation reflects the increased costs, dangers, and legal penalties of the border crossing, which have encouraged migrants to settle for longer periods in the US without the possibility of return or cyclical migration (48, 49).

Service providers frequently addressed the negative mental health impacts of immigrants’ long-term loss of family-based social and support networks. For immigrants, the inability to return to Mexico due to both financial and legal constraints for family events, funerals, and celebrations was a source of on-going sadness and loneliness. Araceli, a 48-year-old from Puebla recalls that during her years without papers she felt very lonely. “When my mother died, they told me from Mexico but I couldn’t go. My grandfather and then my dad, and my uncle who was like a father to me, they all died and I couldn’t go to their funerals. Later I went back and saw their graves. That is my own little piece of loneliness: to have so many years go by without seeing my family.” Participants worked hard to stay in close touch with family via regular phone calls and internet communication, but the physical distance and passage of time frustrate efforts to maintain closeness and many participants bemoaned lost or strained emotional connections with children, siblings, and parents.

Approximately half of study participants reported having little community support in Tucson, and even those with intact nuclear families and strong community ties in Tucson still commonly reported missing the close-knit spontaneous gatherings with extended family in Mexico. Thirty-two-year-old Gregorio who came to the US 4 years ago from Sonora explained that, “Sometimes I am happy here because I have my own family but I also miss my family in Mexico, so I am not totally content. Here my family is just my wife and my kids. There I have my brothers and sisters and my mom and dad, and one also misses them.” For those participants separated from spouses and children, or who have been unable to establish community in Tucson, the feelings of sadness and loneliness are often debilitating and accompanied by depression. Teresa, who remains separated from her husband following his deportation over 1 year ago, described her emotional state: “I have some family here but it’s like it makes no difference, they don’t ask me how I am doing or if we have food, or how my son is. I still feel so alone. I don’t know if I can handle this anymore, I am so lonely here. I don’t go out or talk to anyone – I just go to work and come home.”

### Financial stress

Financial stress was a constant theme during my interviews, with 77.5% of the sample stating that they were only “surviving” or “barely surviving” economically (Table 2). The average monthly income in this study was $1,400 for households consisting of an average of 3.7 people, with several low outliers earning as little as $600 or less (Table 1). Many service providers described a debilitating level of economic stress among Mexican immigrants in Tucson, and observed that this stress had increased over the past 10 years in response both to the economic downturn and the 2007 passage of state legislation requiring all Arizona employers to use the federal E-Verify technology to verify legal status. Many immigrants stated feeling frustrated and impotent against the legal barriers that prevented them from securing more stable and remunerative employment.

Although three-quarters of participants stated that they were financially better off in the US than they had been in Mexico, most reported greater financial stress in the US due to the inflexibility of the American billing system. Referred to ubiquitously as “*los biles*,” monthly bills were a perpetual source of worry for both men and women. The need for adults to work long hours and multiple jobs in order to survive financially put major stressors on families. Women addressed the stress that came from both parents needing to work, and lamented how this left little time for family relaxation and healthy eating habits. Isabella, a 35-year-old participant corroborated the experiences of many other women in this study:

We have more work to do in the house than [the men do]. They go to work and come home and relax. But we don’t get to relax. And so what happens is our health gets worse every day. We can eat all the vegetables and lentils that we want, but if we don’t have peace in our homes and in our minds, then we can’t be healthy. It’s just that the stress of all the rules with everything here. My head hurts here from all the pressure of paying the bills and the go-go-go. We wanted a better life: We got more things, but less life.

Men in the sample commonly emphasized the need to work in order to have positive self-esteem and feel healthy. The majority of men in this sample worked as day laborers and struggled to secure jobs on a daily basis. Many men blamed this lack of job security and stable income for their unhealthy sleep patterns and high levels of generalized stress. Esteban, a 55-year-old undocumented laborer from Sonora explained:

When I don’t work the full 40 hours, I don’t feel like a normal person. A normal person works every day. And I can work – I am a carpenter, I have energy. And I have a nice truck (he points to a truck sitting idly in the parking lot) – I am all ready. So I just feel this stress and a depression that’s like a sadness. The stress gives me a headache so I cannot sleep, and I am up almost all night.

Because the vast majority of sample participants had no economic reserves, many underwent feelings of stress, hopelessness, sadness, and even trauma during periods of transition and hardship. Common stressors included the deportation or incarceration of a spouse, evictions, overcrowded living conditions, temporary homelessness, and reliance on soup kitchens and food banks. Tomás, a 39-year-old father from Sonora described a period of time in which he could not find work for an entire month.

I felt so much stress and *nervios* that my mind got all twisted up. I had to go see the doctor at the clinic because the muscles on one side of my face stopped working completely, so that when I tried to drink water it would just dribble out of my mouth. But the doctor just told me not to worry so much about my life, because that worry became stress and that was damaging my body. And he gave me some pills that I took for awhile.

### Mental health outcomes and access to care

The compounding and cumulative effects of these emotional experiences took a serious toll on the self-reported mental health of this study sample (Table 3). Participants most commonly cited experiencing the emotions of trauma (57.5%), fear (70%), depression (75%), loneliness (77.5%), sadness (82.5%), and stress (87.5%), and most respondents reported suffering from three or more of these emotions. The three-quarters of the sample that stated feeling depressed connected the depression with either long-term declines in well-being or with particularly challenging periods, such as during detention or following the deportation of loved ones. Mental health practitioners I interviewed uniformly reported that the most common mental health issues they witnessed in the immigrant community were depression and anxiety related to economic stress, fear of detention, family separation, social isolation, family stress, and the desert crossing. Practitioners stated that the incidence of depression and anxiety in the immigrant community had increased since the recent economic downturn in Arizona and passage of anti-immigrant legislation. Reverend Raul Trevizo, long-time pastor of Tucson’s largest Mexican-origin Catholic parish, describes what he has seen among his congregants in recent years:

It’s a collective depression, a desperation that is epidemic in the immigrant community. You begin to see all the effects of what could probably be diagnosed as clinical depression. This is very collective; this is not individual. They feel that they are in a country where they are not wanted, they are looked upon suspiciously, they face deportation at any moment, and they are struggling to survive. It’s like a collective post-traumatic stress syndrome.

**Table 3:** **Self-reported emotional condition of participants (%)**.

	Men (*n* **=** 20)	Women (*n***=** 20)
Fear	70	70
Sadness	80	85
Loneliness	70	75
Stress	95	80
Trauma	55	60
Depression	70	85

Strikingly, the vast majority of Mexican immigrants lack access to mental and behavioral health care services that could help mitigate migration-related stress. The passage of House Bill 2008 in 2009 blocked immigrant access to preventive and follow-up mental health care (50). These provisions make it nearly impossible for immigrants to receive behavioral health services unless they are visibly presenting psychotic symptoms and are remanded to crisis response services by police authorities. Even in those dire cases, very little can be done to address on-going issues or secure necessary follow-up care for immigrant patients suffering from psychosis.

Immigrants suffering from lower grade conditions like depression and anxiety were limited to a handful of low-income and free clinics with limited volunteer-staffed counseling programs. However, there are barriers to accessing the scant services available, including limited knowledge about available services and individual willingness to seek emotional support in a community that still has many reservations and negative stereotypes about mental health services (Meza, personal communication). Moreover, even the sliding scale offered by the low-income clinics can be prohibitive for immigrant patients, and many immigrants feel debilitating fear and lack of trust in medical facilities due to having seen Border Patrol policing area hospitals or having known someone who was detained or deported after accessing care (Al-qaraz Ochoa, personal communication). In addition, even those few immigrants who qualify for state Medicare coverage are often afraid to use it because they fear that it will label them as a “public charge” and therefore damage their citizenship applications (Roberts, personal communication).

While access to care is severely curtailed in this community, immigrant participants and services providers alike noted the benefits for those who were able to receive therapy services. Many participants expressed reticence to share openly with friends and family. Leticia explained that talking to the psychologist about her depression related to being undocumented made her feel better “because I really don’t have friends, just my sisters and my husband. And with [the therapist] I can say things I can’t tell my sisters or my husband because they would talk to each other.” Having access to a trained therapist offered an outlet for self-expression that afforded potential emotional and physical healing. Therapist Michelle Humke described a teenager who came to counseling because she lost her ability to walk following the imprisonment of her undocumented mother.

This was a mental health issue that manifested as physical issue – all she knew was that she couldn’t walk, and I said ‘this is all related to the family issue.’ She was in the care of her extended family, and the whole family was traumatized. So I brought them all in and helped them learn how to better verbalize when they were feeling strong emotions and how to talk about it. And this is what helped her walk again.

These results demonstrate the cumulative impact that migration-related emotional suffering has on the overall mental health of immigrants and the potential for expanded mental health services to play a role in healing.

## Discussion

Rather than directly testing the biological impacts of psychosocial stress on the body, this article uses self-reported emotional suffering to place the incidence of such stressors in a broader contextual framework. I use the words of first-generation Mexican immigrants to portray the oppressive nature of living within the structural vulnerabilities at force in their lives and their resulting experiences of emotional suffering and trauma. Because “emotional practices can … be seen as social acts which are significant in revealing the complex interrelationships between the individual and society via the body,” the emotional testimonies presented here offer insight into how Mexican immigrants in southern Arizona experience the daily context of their lives on a body level [(1), p. 396]. These are testimonies to how individuals embody social inequalities, which may in turn help to make sense of existing health disparities.

### An emotional perfect storm

The vast majority of the interview sample for this paper report experiencing multiple negative emotions related to the compounding experiences of childhood adversity, family separation, dangerous border crossings, undocumented status, detention and deportation, and financial stress. Many of these “psychic costs” of migration [(51), p. 297] have been studied previously and linked to self-reported declines in well being and mental health. The pervasive fear of detention and deportation, which dramatically curtails freedom of movement and promotes distrust (12), has been linked to a direct decrease in perceived emotional and physical health status (52). Border crossing and militarization have been linked to the severing of family and social networks and related feelings of isolation and loneliness (11, 53), while deportation itself has been associated with poor self-reported physical and mental health (54). Discrimination and stigma related to immigrant status have been shown to increase self-reported stress and health declines (29). The undocumented community (52), children and separated families (12), and migrant workers (6, 55, 56) have all reported particularly high levels of migration-related stress.

### Science of emotion

Some researchers argue that emotional stress may help explain health disparities patterned across lines of race and socio-economic status. A small but promising body of literature has begun to use bio-marker data to link migration-related stressors to physical health declines (57–59). Says Sternberg: “we are discovering that while feelings don’t directly cause or cure disease, the biological mechanisms underlying them may cause or contribute to disease” (2001: 13). The emotional suffering reported on in this article – namely stress, loneliness, fear, depression, and trauma – implies varied and well-documented biological consequences.

Stress has been so closely linked to widespread and generalized health declines that Sapolsky determines that “chronic stress can be pathogenic” [(60), p. 395]. Deficiencies in social networks have been shown to impact all-cause mortality (61), and loneliness is now understood to produce immune deficiencies and disruptions to cardiovascular functioning and complex cognitive functioning (62). Moreover, the fear response has been linked to the onset of anxiety, depression, and other mental disorders in addition to physical health problems, such as weakened immune function, hypertension, and insulin resistance (63, 64). Likewise, depression is posited to cause dysregulation of the metabolic, immuno-inflammatory, and autonomic and hypothalamic–pituitary–adrenal axis, potentially increasing the incidence of morbidity related to cardiovascular disease, stroke, and obesogenic co-morbidities (65). Lastly, trauma has been shown to disrupt homeostasis via impacts on the brain, endocrine system, and sympathetic nervous system, and “through a physiological domino effect, these changes affect many other body systems, including the cardiovascular system, respiratory system, and muscular system” [(66), p. 52].

### Limitations and strengths

This paper takes a life-course approach to immigrant mental health, incorporating pre-migration factors as well as a complex web of post-migration stressors. According to the theory of “cumulative adversity,” individual hardships cannot be holistically evaluated in isolation, as they both compound and overlap with one another to produce long-term ill effects on physical and mental health (67). The categories I presented in the Section “Results” of this paper are by definition interwoven and iterative, and thus do not break down for neat analysis of the impact of individual factors on immigrant emotional health. Certain categories, namely family separation and undocumented status, undergird all aspects of immigrant experience and are challenging to tease out from their associated impacts on other areas of immigrant’s daily lives. At the same time, the factors I address in this paper are by no means exhaustive of the emotional hardships that may contribute to immigrant’s cumulative toll of adversity over the lifecourse. I only investigated emotional experiences closely linked to migration, and therefore intentionally did not address emotions related to more intimate changes in family dynamics and relationships, such as changing gender roles (68) and role loss (69).

In addition, this paper does not argue that all emotional experiences related to migration are negative. While study participants overwhelmingly reported emotional hardship related to migration, a minority of participants (25%) also stated ways in which immigration had a positive impact on their emotional or physical health. This was especially so for participants who had made a significant life change upon migrating – such as overcoming drug or alcohol addiction, leaving an abusive relationship, or conversion to a new church or religion – or for those who had faced debilitating levels of poverty in Mexico. These examples highlight the possibility for migration to be associated with positive emotions and experiences depending on pre-existing circumstances and individual motivation for change.

## Conclusion

The immigrants and service providers I interviewed indicate that the recent rash of state level exclusionary legislation in Arizona has intensified and compounded emotional hardship in the immigrant community. The state passed English-only legislation in 1987 and 2005, denied undocumented immigrants access to in-state tuition and state scholarship monies through Proposition 300 in 2006, limited immigrant access to state services under HB 2008 in 2009, banned Mexican American Studies from the Tucson public schools in 2010 under HB 2281, and that same year passed SB 1070, requiring immigrants to carry identifying documents at all times and institutionalizing cooperation between police and immigration authorities. The immigrants I interviewed lived with the tangible results of this rash of legislation, including increased risk of detention and deportation, longer family separations, more dangerous border crossings, and restricted access to full employment, education, and medical care. Their emotional reactions to these changes are embodied testimonies to the ways these policies touch individual lives.

In the absence of an overhaul of state and federal immigration policy, the findings presented in this paper indicate several pragmatic solutions that could serve to reduce the incidence and intensity of emotional suffering among Mexican immigrants in southern Arizona. I make the following recommendations in regards to strengthening community support and public safety: (1) expand local networks of support called “*redes de proteccion*” that offer leadership training, community building, and assistance to immigrants in the event of detention or deportation, and thus buffer against related fear, stress, and loneliness; (2) increase access to identification cards currently available from the Mexican Consulate and in discussion by the City of Tucson, which provide migrants with a valid form of identification and thus foster a sense of increased personal security; (3) encourage further dialog between community groups and local police agencies to reduce fear and likelihood of unnecessary non-criminal detention and family separations. In addition, in order to increase immigrant access to mental health services, I make the following recommendations: (1) educate medical personnel regarding the emotional stressors affecting this community and encourage wider application of screening measures for immigrant patients; (2) expand the availability of Spanish language mental health services to screen for mental illness, offer an outlet for immigration-related stress, and aid immigrants in making practical choices to improve their living conditions; and lastly, (3) increase outreach to Spanish speaking immigrants about available health and support services via regularly updated pamphlets and improved communication between service providers.

## Conflict of Interest Statement

The author declares that the research was conducted in the absence of any commercial or financial relationships that could be construed as a potential conflict of interest. The Guest Associate Editor Scott C. Carvajal declares that despite being affiliated to the same institution as author Rebecca Crocker, the review process was handled objectively and no conflict of interest exists.

## References

[1] Powell JL Gilbert T Social theory and emotion: sociological excursions. Int J Sociol Soc Policy (2008) 28(9/10):394–407. 10.1108/01443330810900220

[2] Scheper-Hughes N Death Without Weeping: The Violence of Everyday Life In Brazil. Berkeley, CA: University of California Press (1993).

[3] Green L Lived lives and social suffering: problems and concerns in medical anthropology. Med Anthropol Q (1998) 12(1):3–7. 10.1525/maq.1998.12.1.3

[4] Quesada J Hart LK Bourgois P. Structural vulnerability and health: Latino migrant laborers in the United States. Med Anthropol (2011) 30(4):339–62. 10.1080/01459740.2011.576725 21777121 PMC3146033

[5] Castañeda H Holmes S Madrigal D De Trinidad Young M Beyeler N Quesada J Immigration as a social determinant of health. Annu Rev Public Health (2014) 36:375–92. 10.1146/annurev-publhealth-032013-182419 25494053

[6] Carvajal SC Rosales C Rubio-Goldsmith R Sabo S Ingram M McClelland DJ The border community and immigration stress scale: a preliminary examination of a community responsive measure in two southwest samples. J Immigr Minor Health (2012) 15(2):427–36. 10.1007/s10903-012-9600-z 22430894 PMC4431619

[7] Martínez DE Slack J Heyman J Report. Migrant Mistreatment While in U.S. Custody. Part I of Bordering on Criminal: the Routine Abuse of Migrants in the Removal System. Washington, DC: American Immigration Council’s Immigration Policy Center (2013).

[8] Sabo S Shaw S Ingram M Teufel-Shone N Carvajal S de Zapien JG Everyday violence, structural racism and mistreatment at the US-Mexico border. Soc Sci Med (2014) 109:66–74. 10.1016/j.socscimed.2014.02.005 24705336

[9] de Haymes MV Martone J Munoz L Grossman S Family cohesion and social support: protective factors for acculturation stress among low-acculturated Mexican migrants. J Poverty (2011) 15(4):403–26. 10.1080/10875549.2011.615608

[10] Chavez L Shadowed Lives: Undocumented Immigrants in American Society. Fort Worth, TX: Harcourt Brace Jovanovich College Publishers (1992).

[11] Talavera V Nuñez GG Heyman J Deportation in the U.S.-Mexico borderlands: anticipation, experience, and memory. In: de Genova N Peutz NM, editors. The Deportation Regime: Sovereignty, Space, and the Freedom of Movement. Durham, NC: Duke University Press (2010). p. 166–95.

[12] Dreby J The burden of deportation on children in Mexican immigrant families. J Marriage Fam (2012) 74(4):829–45. 10.1111/j.1741-3737.2012.00989.x

[13] Holmes SM Fresh Fruit, Broken Bodies: Migrant Farmworkers in the United States (Vol. 27). Berkeley, CA: University of California Press (2013).

[14] Napolitano Quayson V Social suffering and embodied states of male transnational migrancy in San Francisco, California. Global Stud Power Cult (2005) 12(3):335–62.

[15] Krieger N Theories for social epidemiology in the 21st century: an ecosocial perspective. Int J Epidemiol (2001) 30(4):668 10.1093/ije/30.4.668 11511581

[16] Braveman PA Cubbin C Egerter S Williams DR Pamuk E. Socioeconomic disparities in health in the United States: what the patterns tell us. Am J Public Health (2010) 100(S1):S186–96. 10.2105/AJPH.2009.166082 20147693 PMC2837459

[17] Dressler WW Oths KS Gravlee CC Race and ethnicity in public health research: models to explain health disparities. Annu Rev Anthropol (2005) 34:231–52. 10.1146/annurev.anthro.34.081804.120505

[18] Gravlee CC. How race becomes biology: embodiment of social inequality. Am J Phys Anthropol (2009) 139:47–57. 10.1002/ajpa.20983 19226645

[19] Krieger N. Social inequalities and health – embodying inequality: a review of concepts, measures, and methods for studying health consequences of discrimination. Int J Health Serv (1999) 29(2):295. 10.2190/M11W-VWXE-KQM9-G97Q 10379455

[20] Lock M Cultivating the body: anthropology and epistemologies of bodily practice and knowledge. Annu Rev Anthropol (1993) 22:133–55. 10.1146/annurev.an.22.100193.001025

[21] Lock M. The tempering of medical anthropology: troubling natural categories. Med Anthropol Q (2001) 15(4):478–92. 10.1525/maq.2001.15.4.478 11794872

[22] Fullilove MT. Psychiatric implications of displacement: contributions from the psychology of place. Am J Psychiatry (1996) 153(12):1516. 10.1176/ajp.153.12.1516 8942445

[23] Cole SW. Elevating the perspective on human stress genomics. Psycho­neuroendocrinology (2010) 35(7):955–62. 10.1016/j.psyneuen.2010.06.008 20630660 PMC2917592

[24] Sternberg E The Balance Within: The Science Connecting Health and Emotions. New York, NY: W.H. Freeman & Co (2001).

[25] Van der Kolk BA The Body Keeps the Score: Brain, Mind, and Body in the Healing of Trauma. New York, NY: Viking Penguin (2014).

[26] Marmot M Social determinants of health inequalities. Lancet (2005) 365(9464):1099–104. 10.1016/S0140-6736(05)74234-3 15781105

[27] Wilkinson R Pickett K The Spirit Level: Why Greater Equality Makes Societies Stronger. New York, NY: Bloomsbury Press (2013).

[28] Hunt LM Schneider S Comer B. Should “acculturation” be a variable in health research? A critical review of research on US Hispanics. Soc Sci Med (2004) 59(5):973–86. 10.1016/j.socscimed.2003.12.009 15186898

[29] Viruell-Fuentes E. Beyond acculturation: immigration, discrimination, and health research among Mexicans in the United States. Soc Sci Med (2007) 65(7):1524–35. 10.1016/j.socscimed.2007.05.010 17602812

[30] Hertzman C Boyce T. How experience gets under the skin to create gradients in developmental health. Annu Rev Public Health (2010) 31(1):329–47. 10.1146/annurev.publhealth.012809.103538 20070189

[31] Passel JS Cohn D Unauthorized Immigrant Totals Rise in 7 States, Fall in 14: Decline in Those From Mexico Fuels Most State Decreases. Washington, DC: Pew Research Center’s Hispanic Trends Project (2014).

[32] Boyce G Launius S Normalizing noncompliance: militarization and resistance in southern Arizona. Bad Subj (2011) 81 Available from: bad.eserver.org/issues/2011/81/boyce-launius.htm

[33] Cadava GL Standing on Common Ground: The Making of a Sunbelt Borderland. Cambridge, MA: Harvard University Press (2013).

[34] Hardy LJ Getrich CM Quezada JC Guay A Michalowski RJ Henley E. A call for further research on the impact of state-level immigration policies on public health. Am J Public Health (2012) 102(7):1250–4. 10.2105/AJPH.2011.300541 22594736 PMC3477996

[35] Muhib FB Lin LS Stueve A Miller RL Ford WL Johnson WD A venue-based method for sampling hard-to-reach populations. Public Health Rep (2001) 116(90001):216–22. 10.1093/phr/116.S1.216 11889287 PMC1913675

[36] Singer B Ryff CD Carr D Magee WJ Linking life histories and mental health: a person-centered strategy. Sociol Methodol (1998) 28:1–52. 10.1111/0081-1750.00041

[37] Spallek J Zeeb H Razum O. What do we have to know from migrants’ past exposures to understand their health status? A life course approach. Emerg Themes Epidemiol (2011) 8(1):6. 10.1186/1742-7622-8-6 21843354 PMC3169503

[38] Horton SB Barker JC. A Latino oral health paradox? Using ethnography to specify the biocultural factors behind epidemiological models. NAPA Bull (2010) 34(1):68–83. 10.1111/j.1556-4797.2010.01052.x 21132097 PMC2995897

[39] Gálvez A Patient Citizens, Immigrant Mothers: Mexican Women, Public Prenatal Care, and the Birth Weight Paradox. New Brunswick, NJ: Rutgers University Press (2011).

[40] Martinez AD. Reconsidering acculturation in dietary change research among Latino immigrants: challenging the preconditions of US migration. Ethn Health (2013) 18(2):115–35. 10.1080/13557858.2012.698254 22731980 PMC3536930

[41] Van Hook J Baker E Altman CE Frisco ML. Canaries in a coalmine: immigration and overweight among Mexican-origin children in the US and Mexico. Soc Sci Med (2012) 74(2):125–34. 10.1016/j.socscimed.2011.10.007 22153862 PMC3259272

[42] Thurston WE Vissandjée B. An ecological model for understanding culture as a determinant of women’s health. Crit Public Health (2005) 15(3):229–42. 10.1080/0958159050037212111988134

[43] Boeije H Analysis in Qualitative Research. Los Angeles: SAGE (2010).

[44] Green L. The nobodies: neoliberalism, violence, and migration. Med Anthropol (2011) 30(4):366–85. 10.1080/01459740.2011.576726 21777123

[45] Martínez DE Reineke RC Rubio-Goldsmith R Anderson BE Hess GL Parks BO A Continued Humanitarian Crisis at the Border: Undocumented Border Crosser Deaths Recorded by the Pima County Office of the Medical Examiner, 1990-2012. Report. The Binational Migration Institute. Tucson, AZ: University of Arizona (2013).

[46] Cremniter D Baudin M Roujeau JC Prost C Consoli SG Francés C Stressful life events as potential triggers of pemphigus. Arch Dermatol (1998) 134:1486–7. 10.1001/archderm.134.11.1486 9828894

[47] Slack J Martínez DE Whiteford S Peiffer E In the Shadow of the Wall: Family Separation, Immigration Enforcement and Security. Report. The Center for Latin American Studies. Tucson, AZ: University of Arizona (2013).

[48] Cornelius WA Impacts of the 1986 US immigration law on emigration from rural Mexican sending communities. Popul Dev Rev (1989) 15(4):689–705. 10.2307/1972595

[49] Dreby J Divided by Borders: Mexican Migrants and Their Children. Berkeley, CA: University of California Press (2010).

[50] Armin J Reineke R Expanding Vulnerability: Health Care, Well-Being, and Arizona’s Immigration Policies. Access Denied: A Conversation on Un/authorized Im/migration and Health (2010). Available from: https://accessdeniedblog.wordpress.com/2010/07/11/expanding-vulnerability-health-care-well-being-and-arizona%E2%80%99s-immigration-policies-%E2%80%93-julie-armin-robin-reineke/

[51] Massey DS Riosmena F. Undocumented migration from Latin America in an era of rising U.S. enforcement. Ann Am Acad Pol Soc Sci (2010) 630(1):294–321. 10.1177/0002716210368114 20824109 PMC2932631

[52] Cavazos-Rehg PA Zayas LH Spitznagel EL. Legal status, emotional well-being and subjective health status of Latino immigrants. J Natl Med Assoc (2007) 99(10):1126–31. 17987916 PMC2574408

[53] Munoz-Laboy M Hirsch JS Quispe-Lazaro A. Loneliness as a sexual risk factor for male Mexican migrant workers. Am J Public Health (2009) 99(5):802–10. 10.2105/AJPH.2007.122283 19299684 PMC2667832

[54] No More Deaths. The Health Impact of Deportation Fact Sheet (2012). Available from: http://forms.nomoredeaths.org/wp-content/uploads/2014/10/DIS-Fact-Sheet-Dec2012-final.pdf

[55] Magaña CG Hovey JD. Psychosocial stressors associated with Mexican migrant farmworkers in the midwest United States. J Immigr Health (2003) 5(2):75–86. 10.1023/A:1022955825650 14512761

[56] Smith-Nonini S. The illegal and the dead: are Mexicans renewable energy? Med Anthropol (2011) 30(5):454–74. 10.1080/01459740.2011.577045 21916680

[57] D’Alonzo KT Johnson S Fanfan D. A biobehavioral approach to understanding obesity and the development of obesogenic illnesses among Latino immigrants in the United States. Biol Res Nurs (2012) 14(4):364–74. 10.1177/1099800412457017 22923710 PMC6334848

[58] Holmes LM Marcelli EA. Neighborhoods and systemic inflammation: high CRP among legal and unauthorized Brazilian migrants. Health Place (2012) 18:683–93. 10.1016/j.healthplace.2011.11.006 22401803 PMC3319645

[59] McClure HH Snodgrass JJ Martínez CR Eddy JM Integrating biomarkers into research with Latino immigrants in the United States. Adv Anthropol (2013) 3(2):112–20. 10.4236/aa.2013.32015

[60] Sapolsky RM Social status and health in humans and other animals. Annu Rev Anthropol (2004) 33:393 10.1146/annurev.anthro.33.070203.144000

[61] Berkman LF Glass T Brissette I Seeman TE. From social integration to health: Durkheim in the new millennium. Soc Sci Med (2000) 51(6):843–57. 10.1016/S0277-9536(00)00065-4 10972429

[62] Cacioppo JT Patrick W Loneliness: Human Nature and the Need for Social Connection. New York, NY: W.W. Norton & Co (2008).

[63] Rodrigues SM LeDoux JE Sapolsky RM. The influence of stress hormones on fear circuitry. Annu Rev Neurosci (2009) 32(1):289–313. 10.1146/annurev.neuro.051508.135620 19400714

[64] Sapolsky RM Romero LM Munck AU. How do glucocorticoids influence stress responses? Integrating permissive, suppressive, stimulatory, and preparative actions. Endocr Rev (2000) 21:55–89. 10.1210/er.21.1.55 10696570

[65] Penninx BW Milaneschi Y Lamers F Vogelzangs N. Understanding the somatic consequences of depression: biological mechanisms and the role of depression symptom profile. BMC Med (2013) 11(1):129. 10.1186/1741-7015-11-129 23672628 PMC3661358

[66] Solomon EP Heide KM The biology of trauma: implications for treatment. J Interpers Violence (2005) 20(1):51–60. 10.1177/0886260504268119 15618561

[67] Seery MD Holman EA Silver RC. Whatever does not kill us: cumulative lifetime adversity, vulnerability, and resilience. J Pers Soc Psychol (2010) 99(6):1025–41. 10.1037/a0021344 20939649

[68] Hirsch JS A Courtship After Marriage: Sexuality and Love in Mexican Transnational Families. Berkeley, CA: University of California Press (2003).

[69] Fadiman A The Spirit Catches You and You Fall Down: A Hmong Child, Her American Doctors, and The Collision of Two Cultures. New York, NY: Farrar, Straus & Giroux (2012).

